# Impaired Glucose Metabolism in People with Extremely Elevated High-Density Lipoprotein Cholesterol and Low Alcohol Consumption: Results of the Kanagawa Investigation of Total Checkup Data from the National Database-3 (KITCHEN-3)

**DOI:** 10.3390/jcm8111825

**Published:** 2019-11-01

**Authors:** Kei Nakajima, Ryoko Higuchi

**Affiliations:** 1 School of Nutrition and Dietetics, Faculty of Health and Social Services, Kanagawa University of Human Services, 1-10-1 Heisei-cho, Yokosuka, Kanagawa 238-8522, Japan; higuchi-nk3@kuhs.ac.jp; 2 Department of Endocrinology and Diabetes, Saitama Medical Center, Saitama Medical University, 1981 Kamoda, Kawagoe, Saitama 350-8550, Japan; 3 Graduate School of Health Innovation, Kanagawa University of Human Services, Research Gate Building Tonomachi 2-A, 3-25-10 Tonomachi, Kawasaki, Kanagawa 210-0821, Japan

**Keywords:** extremely high, fasting plasma glucose, HbA_1c_, HDL, nondrinker

## Abstract

Background: Recently, we have shown that extremely high high-density lipoprotein cholesterol (HDL-C), which was observed mostly in heavy drinkers, was associated with the incidence of diabetes. However, the observed association was influenced by the consumption of alcohol. Furthermore, it is unknown whether impaired glucose metabolism exists in people with extremely high HDL-C, regardless of their alcohol consumption. Therefore, we addressed this issue in people who did not have a habit of drinking alcohol. Methods: In this community-based cross-sectional study, we included 177,034 participants (40–74 years old) who reported being nondrinkers. We investigated levels of HbA_1c_, fasting plasma glucose (FPG), HDL-C, and clinical parameters according to 11 levels of HDL-C concentration from 20 to 120 mg/dL or over. Results: A total of 6112 participants with HDL-C ≥ 100 mg/dL (3.5%) showed a better lipid profile, higher prevalence amongst women, more habitual exercise, a lower prevalence of smoking, and lower body mass index (BMI). Compared with an HDL-C of 70–79 mg/dL, HDL-C ≤ 69 mg/dL (except an HDL-C of 20–29 mg/dL) and HDL-C ≥ 90 mg/dL were significantly associated with a high HbA_1c_ of ≥6.0%, independently of confounding factors. This finding was distinctly demonstrated in women. Similar trends were observed when high HbA_1c_ was replaced with high FPG (≥110 mg/dL). Conclusions: Our study demonstrated that impaired glucose metabolism may exist in people with extremely high HDL-C and who hardly drink alcohol.

## 1. Introduction

Recently, doubt has been cast on the protective effect of very elevated high-density lipoprotein cholesterol (HDL-C) against all-cause mortality and cardiovascular disease (CVD) in particular [1,2]. We recently showed that people with extremely high HDL-C (≥110 mg/dL) have an increased risk for diabetes, which is a strong risk factor for CVD [3]. However, the observed association between extremely high HDL-C and the incidence of diabetes may be complicated because high HDL-C concentrations can be attributed to several factors, such as genomic [4] and particularly lifestyle factors like habitual exercise [5] and heavy alcohol consumption [6]. In our previous cohort study, when participants were restricted to those who hardly drank alcohol, a significant association between extremely high HDL-C and incident diabetes was not observed [3], which suggests that alcohol consumption may have a pivotal role in these associations. Nevertheless, the duration of our previous study was relatively short (6 years), which may be inadequate to evaluate the incidence of overt diabetes in nondrinkers because people who do not habitually drink alcohol may pay greater attention to their general health.

In this study, to elucidate the fundamental association between extremely high HDL-C and impaired glucose metabolism without the effect of alcohol consumption, we investigated whether impaired glucose metabolism rather than overt diabetes existed in people with extremely high HDL-C who hardly drink alcohol in a community-based cross-sectional study of apparently healthy Japanese people.

## 2. Methods

We conducted a composite multidisciplinary study involving the secondary use of mandatory health check data in Japan, utilizing the Kanagawa Investigation of the Total Checkup Data from the National Database (KITCHEN), to elucidate the factors primarily associated with cardiometabolic diseases. The overall study concept and design has been described elsewhere [7]. The current study included any individual who underwent this specific health check and who was living in Kanagawa Prefecture. The study protocol was approved by the ethics committee of the Kanagawa University of Human Services (10–43) and the Ministry of Health, Labor, and Welfare of Japan (No. 121).

This cross-sectional study used data collected from 1.8 million people who attended health checks between April 2014 and March 2015. Participants undergoing pharmacotherapy for dyslipidemia and/or diabetes were not included. However, some included participants had undergone pharmacotherapy for hypertension and some had a history of CVD. All these conditions were digitally recorded as questionnaire responses.

The frequency and amount of alcohol consumption were evaluated with two survey questions: (1) How often do you drink alcohol? (Three response options: every day, occasionally, and hardly/cannot drink). (2) How much do you drink a day, in terms of glasses of refined sake? (A glass (180 mL) of refined sake is equivalent to a medium bottle (500 mL) of beer, 80 mL of shochu (alcohol content 35%), a glass (double, 60 mL) of whiskey, and two glasses (240 mL) of wine). According to the response, the quantity of alcohol consumption was classified into four degrees: (1) less than 180 mL (<23 g ethanol), (2) over 180–360 mL (23–45 g ethanol), (3) over 360–540 mL (46–68 g ethanol), and (4) over 540 mL (≥69 g ethanol). These questions were made by Japanese Ministry of Health, Labour and Welfare [7]. Participants were restricted to those who responded that they hardly or did not consume alcohol and those who consumed <23 g ethanol/day (meeting both criteria). Consequently, a total of 177,034 people (57,789 men and 119,245 women) aged 40–74 years satisfied these criteria and were enrolled in the study.

As in our previous project [7], participants were categorized into the age groups of 40–44, 45–49, 50–54, 55–59, 60–64, 65–69, and 70–74 years. Participants’ precise ages were unknown to prevent identification of specific individuals. However, to evaluate participant age as a single numeric value, we transformed the above age groups into substituted ages (s-age), corresponding with the median of each age group (42, 47, 52, 57, 62, 67, and 72 years, respectively).

Participants were also classified into 11 categories with intervals of 10 mg/dL of HDL-C according to HDL-C concentration: 20–29, 30–39, 40–49, 50–59, 60–69, 70–79, 80–89, 90–99, 100–109, 110–119, and ≥120 mg/dL. Though a lower limit for circulating HDL-C, such as 40 mg/dL, is used as a guideline for the prevention of cardiovascular disease in American and Japanese guidelines [8,9], an optimal range of HDL-C has not fully been argued for regarding the prevention of other diseases. In our previous study [7], the provisional reference of HDL-C was considered to be 80–89 mg/dL because the relative risk for diabetes after six years was the lowest in subjects with an HDL-C of 80–89 mg/dL. As such, in this study, we defined an HDL-C of 80–89 mg/dL as a reference HDL-C at the beginning of the analysis.

### 2.1. Measurements

Body mass index (BMI) was calculated as mass (kg) divided by the square of height (m^2^). Biochemical measurements were performed automatically using standard methods. Serum low-density lipoprotein cholesterol (LDL-C), HDL-C, and triglyceride (TG) concentrations were measured automatically, mainly (approximately 85% of samples) spectrophotometrically using a direct, non-precipitation method; concentrations in the remaining samples were measured using other methods. HbA_1c_ was mainly measured using an immunoassay (65%) or high-performance liquid chromatography (17%) [7]. Fasting plasma glucose (FPG) was primarily measured (about 54% of samples) spectrophotometrically or potentiometrically (about 30% of samples).

High HbA_1c_ and high FPG were defined as HbA_1c_ ≥ 42 mmol/mol (≥6.0%) and FPG ≥ 110 mg/dL (6.1 mmol/L), respectively, based on the criteria of prediabetes [10].

### 2.2. Statistical Analysis

Data are expressed as mean ± SD or median (interquartile range, IQ). Continuous and categorical variables were compared between HDL-C groups using ANOVA, Dunnett’s test (HbA_1c_ and FPG), or Cochran–Armitage tests. Correlations of HDL-C and other variables were tested using Pearson’s correlation. A logistic regression model was used to evaluate associations between HDL-C concentration and the prevalence of high HbA_1c_ and high FPG, without or with adjustment for confounding factors, yielding adjusted odd ratios (ORs) and 95% confidence intervals (CIs). The effect of interactions between sex, smoking, exercise, and BMI was also added to the model. Subsequently, the effect of combined variables (sex, smoking, exercise, and BMI) was adjusted in the model. Statistical analysis was performed using SAS Enterprise Guide (SAS-EG 7.1) in SAS, version 9.4 (SAS Institute, Cary, NC, USA). A *p*-value < 0.05 was considered to indicate statistical significance.

## 3. Results

Table 1 shows the participant characteristics, classified according to the 11 categories of HDL-C concentration. There were 6112 participants with HDL-C ≥ 100 mg/dL (3.5% of the total), and nearly 95% were women. The s-age, prevalence of women, regular exercise, and physical activity more than 1 h per day were higher in the higher HDL-C groups than in the lower groups; however, BMI, blood pressures, TG, LDL-C, HbA_1c_, and FPG were lower (ANOVA or Cochran–Armitage test; all *p* < 0.0001). Pharmacotherapy for hypertension, past history of CVD, current smoking, high HbA_1c_, and high FPG were less prevalent in the higher HDL-C groups (Cochran–Armitage test; all *p* < 0.0001). However, the prevalence of high HbA_1c_ was significantly higher in the highest HDL-C category, ≥ 120 mg/dL, than in the group with HDL-Cs of 80–89 mg/dL (Dunnett’s test); this also applied for women. We did not divide participants according to sex because of the small number of participants with a high FPG at the ends of the range of HDL-C levels (*n* = 38 for the lowest HDL-C group and 41 for the highest HDL-C group). When the participants were divided between men (*n* = 57,789) and women (*n* = 119,245), similar trends were observed in most variables in men and women (Tables S1 and S2). However, the correlation between s-age and HDL-C differed between men and women; the correlation was positive in men (*r* = 0.05, *p* < 0.0001), whereas it was negative in women (*r* = −0.02, *p* < 0.0001). In addition, the proportion of subjects with HDL-C ≥ 100 mg was very rare (<1.0%) in men, whereas the prevalence was around 5% in women.

Table 2 shows the results of the logistic regression analysis. Compared with participants who had an HDL-C of 80–89 mg/dL, those with HDL-C ≤ 79 mg/dL and HDL-C ≥ 110 mg/dL were significantly associated with high HbA_1c_ (model 1); this was nearly unchanged after adjustment for age and sex (model 2). However, after fully adjusting the remaining confounders, including smoking, habitual exercise, and BMI, an HDL-C of 30–69 mg/dL and HDL-C ≥ 90 mg/dL were significantly associated with high HbA_1c_ (Model 3), as compared with an HDL-C of 70–79 mg/dL. An HDL-C of 20–29 mg/dL was inversely associated with high HbA_1c_. Further adjustment for the effect of the interaction between sex, BMI, exercise, and smoking status did not change these associations (data not shown). Furthermore, additional adjustment for the combined effect of relevant variables (sex, BMI, exercise, and smoking status) did not largely change the associations (model 4).

When participants were divided according to sex, associations (HDL-C ≥ 110 mg/dL) and high HbA_1c_ were distinctly demonstrated in women, whereas a significant inverse association between an HDL-C of 20–29 mg/dL and high HbA_1c_ was found in men. Similar associations were observed when high HbA_1c_ was replaced with high FPG ≥ 110 mg/dL.

## 4. Discussion

It has been shown that HDL particles may exert anti-diabetogenic functions by enhancing glucose-stimulated insulin secretion and glucose uptake into skeletal muscle, adipose tissue, and the liver [11,12].

However, the present study suggests that even among nondrinkers, impaired glucose metabolism can exist in people with extremely high HDL-C, regardless of sex. Although HbA_1c_ was inversely correlated with HDL-C in a rough evaluation, strictly speaking, the relationship between the odds ratios for high HbA_1c_ and high FPG and the HDL-C categories showed a right–left inverted J-shaped curves with the bottom at an HDL-C of 80–89 mg/dL or 70–79 mg/dL. Notably, associations between extremely high HDL-C and impaired glucose metabolism were strengthened after an adjustment for relevant confounding factors, whereas associations between low HDL-C and impaired glucose metabolism were weakened or inverted. Current findings reflect the substantial effects of confounding factors, including BMI, smoking, and habitual exercise, on the observed associations, although further adjustment for the interaction between variables and the combined effect of variables did not change these associations. Clear associations between extremely high HDL-C and impaired glucose metabolism were observed in women but not men. The most plausible explanation for this is that the actual number of men was reduced in groups with extremely high HDL-C (≥100 mg/dL) and the statistical power was weakened; the initial exclusion of individuals who drank alcohol would generally affect more men than women [6].

For the last decade, several trials to raise HDL-C concentrations pharmacologically by inhibiting the cholesteryl ester transfer protein were mostly unsuccessful in the prevention of CVD [13,14,15], suggesting that some factors beyond HDL-C concentration per se may be responsible for the increased risks of CVD. In the current results, the underlying mechanism between extremely high HDL-C and impaired glucose metabolism, a well-known risk for CVD, remains unknown, although participants with extremely high HDL-C levels had favorable profiles for weight, lipids, exercise, and smoking. Likely contributors to the observed associations can include rare dysfunctional HDL-C that exerts an impairing glucose metabolism, if any, and other factors, such as genetic factors (*CETP, APOA1, LCAT*, and so on) [4], and impaired hormones relating to glucose metabolism, such as reduced insulin secretion and increased counterregulatory hormones (adrenaline, glucagon, thyroid hormones, growth hormone, and cortisol, among others). Particularly concerning adrenaline, a catecholamine hormone secreted by the adrenal medulla, Okamura et al. [16] have shown that BMI was significantly increased, and serum HbA_1c_ and HDL-C were decreased after an adrenalectomy in patients with pheochromocytoma. Similar findings were observed after treatment of pheochromocytoma in another study by Bosanska et al. [17], albeit a significant reduction in HDL-C was not observed. Moreover, it is possible that an elevated HDL-C in patients with pheochromocytoma could be considered an indirect effect of catecholamines-induced thermogenic activation of adipocytes [18,19].

In this study, higher rates of habitual exercise and physical activity in conjunction with relatively low body weight (low BMI < 20.0 kg/m^2^ for women) were observed in the groups of higher HDL-C (≥100 mg/dL). These conditions might increase oxidative stress and chronic inflammation owing to concomitant reduced muscle mass and subtle malnutrition, which in turn may lead to impaired reverse cholesterol transport [20,21], and consequently, to elevated HDL-C. In conjunction with this, it has been shown that underweight or lean people often have diabetes [22,23].

Additionally, this study showed that very low HDL-C (20–29 mg/dL) was positively associated with an impaired glucose metabolism, which was inverted after adjustment for the remaining confounders including smoking and BMI. This suggests the possibility that smoking and BMI may substantially influence the association between the lowest HDL-C levels and impaired glucose metabolism.

## 5. Conclusions

In conclusion, impaired glucose metabolism can exist in people with extremely high HDL-C who have very low alcohol consumption, irrespective of overall favorable profiles. The present findings warrant further investigation.

## Figures and Tables

**Table 1:** Characteristics of participants according to HDL-C category.

HDL-C Category (mg/dL)	20–29	30–39	40–49	50–59	60–69	70–79	80–89	90–99	100–109	110–119	≥120
*n* (% of total)	239 (0.1)	6626 (3.7)	24,770 (14.0)	37,770 (21.3)	40,245 (22.7)	32,046 (18.1)	19,494 (11.0)	9732 (5.5)	3981 (2.3)	1383 (0.8)	748 (0.4)
s-Age (years)	55.2 ± 10.9	54.7 ± 10.6	55.0 ± 10.6	55.7 ± 10.8	55.9 ± 10.8	56.1 ± 10.7	56.3 ± 10.4	56.5 ± 10.2	56.8 ± 9.9	57.4 ± 9.7	58.3 ± 9.6
Women, *n* (%)	26 (10.9)	1233 (18.6)	8138 (32.9)	20,586 (54.5)	29,659 (73.7)	27,233 (85.0)	17,622 (90.4)	8977 (92.2)	3758 (94.4)	1305 (94.4)	708 (94.7)
BMI (kg/m^2^)	24.9 ± 2.9	24.6 ± 2.8	24.0 ± 2.9	23.0 ± 3.0	21.9 ± 2.9	21.1 ± 2.7	20.5 ± 2.5	20.1 ± 2.4	19.8 ± 2.4	19.5 ± 2.2	19.5 ± 2.5
SBP (mmHg)	123 ± 17.4	123 ± 16.2	122 ± 16.7	121 ± 17.3	119 ± 17.4	118 ± 17.5	117 ± 17.5	117 ± 17.4	117 ± 17.6	117 ± 17.5	117 ± 17.6
DBP (mmHg)	75.8 ± 11.5	76.3 ± 11.1	75.7 ± 11.3	74.2 ± 11.4	72.4 ± 11.2	71.4 ± 11.1	70.9 ± 11.1	70.7 ± 10.9	70.6 ± 11.0	70.7 ± 10.9	71.1 ± 10.9
HDL-C (mg/dL)	27 ± 1.9	36 ± 2.4	45 ± 2.8	55 ± 2.9	64 ± 2.9	74 ± 2.9	84 ± 2.8	94 ± 2.8	104 ± 2.8	114 ± 2.8	130 ± 11.1
LDL-C (mg/dL)	105 ± 34	128 ± 34	137 ± 32	136 ± 33	132 ± 32	129 ± 31	128 ± 30	127 ± 30	127 ± 30	129 ± 31	125 ± 33
TG, IQ (mg/dL)	236 (146–360)	169 (122–238)	125 (91–170)	96 (71–129)	78 (59–103)	68 (53–89)	63 (49–80)	59 (47–75)	56 (45–71)	56 (45–70)	55 (44–70)
HbA_1c_ (%)	5.96 ± 0.60 ^a^	5.99 ± 0.54 ^a^	5.95 ± 0.48 ^a^	5.89 ± 0.43 ^a^	5.84 ± 0.37 ^a^	5.82 ± 0.33 ^a^	5.81 ± 0.32	5.81 ± 0.31	5.82 ± 0.30	5.83 ± 0.28	5.86 ± 0.29 ^a^
High HbA_1c_, *n* (%)—Total	84 ^a^ (35.2)	2531 ^a^ (38.2)	8555 ^a^ (34.5)	11,224 ^a^ (29.7)	10,339 ^a^ (25.7)	7376 (23.0)	4302 (22.1)	2144 (22.0)	895 (22.5)	337 (24.4)	207 ^a^ (27.7)
High HbA_1c_, *n* (%)—Men	73 ^a^ (34.3)	2021 ^a^ (37.5)	5478 ^a^ (32.9)	4761 ^a^ (27.7)	2613 (24.7)	1056 (21.9)	411 (22.0)	176 (23.3)	67 (30.0)	15 (19.2)	10 (25.0)
High HbA_1c_, *n* (%)—Women	11 (42.3)	510 ^a^ (41.4)	3077 ^a^ (37.8)	6463 ^a^ (31.4)	7726 ^a^ (26.1)	6320 (23.2)	3891 (22.1)	1968 (21.9)	828 (22.0)	322 (24.7)	197 ^a^ (27.8)
FPG (mg/dL)	99.5 ± 20.1 ^a^	98.1 ± 15.7 ^a^	96.5 ± 14.0 ^a^	94.2 ± 12.5 ^a^	92.3 ± 11.1 ^a^	91.1 ± 10.0 ^a^	90.4 ± 9.6	90.6 ± 9.5	90.5 ± 9.4	90.6 ± 9.3	91.4 ± 10.2
High FPG, *n* (%)—Total	38 ^a^ (15.9)	914 ^a^ (13.8)	2708 ^a^ (10.9)	2938 ^a^ (7.8)	2104 ^a^ (5.2)	1252 ^a^ (3.9)	617 (3.2)	302 (3.1)	127 (3.2)	49 (3.5)	41 (5.5)
Pharmacotherapy for hypertension, *n* (%)	35 (14.6)	1071 (16.2)	3818 (15.4)	5244 (13.9)	4505 (11.2)	3130 (9.8)	1711 (8.8)	791 (8.1)	292 (7.3)	97 (7.0)	73 (9.8)
CVD, *n* (%)	— ^b^	193 (2.9)	671 (2.7)	862 (2.3)	812 (2.0)	641 (2.0)	378 (1.9)	170 (1.8)	68 (1.7)	18 (1.3)	14 (1.9)
Current smoking, *n* (%)	109 (45.6)	2588 (39.1)	6771 (27.3)	6295 (16.7)	4194 (10.4)	2172 (6.8)	1001 (5.1)	381 (3.9)	148 (3.7)	46 (3.3)	20 (2.7)
Habitual exercise, *n* (%) ^c^	57 (23.9)	1509 (22.8)	6263 (25.3)	10,555 (28.0)	11,644 (28.9)	9609 (30.0)	6054 (31.1)	3227 (33.2)	1286 (32.3)	512 (37.0)	287 (38.4)
Mild to moderate physical activity, *n* (%) ^d^	98 (42.1)	2418 (37.1)	10,104 (41.4)	16,781 (45.0)	18,949 (47.6)	15,873 (50.0)	10,067 (52.2)	5291 (54.8)	2171 (55.0)	780 (57.0)	434 (58.5)

^a^ Statistically significant difference in HbA_1c_, high HbA_1c_, FPG, and high FPG using Dunnett’s test compared with the 80–89 mg/dL HDL-C group. Dunnett’s test was conducted after the case was numbered as 1 and non-case as 0. ^b^ Not expressed because of the small number (<10), which could affect participants’ confidentiality. ^c^ Habitual exercise to a light sweat for over 30 min per session, twice weekly. ^d^ Physical activity (walking, and so on) more than 1 h per day (available *n* = 175,044). BMI—Body mass index, SBP—Systolic blood pressure, DBP—Diastolic blood pressure, HDL-C—High-density lipoprotein cholesterol, LDL-C—Low-density lipoprotein cholesterol, TG—Triglyceride, IQ—interquartile range FPG—Fasting plasma glucose, CVD—Cardiovascular disease. High HbA_1c_—HbA_1c_ ≥ 42 mmol/mol (≥6.0%); high FPG—FPG ≥ 110 mg/dL (6.1 mmol/L).

**Table 2:** Odds ratios (95% confidence intervals) of HDL cholesterol (HDL-C) categories for high HbA_1c_ and high fasting plasma glucose (FPG).

HDL-C (mg/dL)	20–29	30–39	40–49	50–59	60–69	70–79	80–89	90–99	100–109	110–119	≥120
High HbA_1c_											
Total											
Model 1	1.91 ***	2.18 ***	1.86 ***	1.49 ***	1.22 ***	1.06 *	1 (ref)	1	1.02	1.14 *	1.35 **
	(1.46–2.50)	(2.06–2.32)	(1.79–1.95)	(1.43–1.56)	(1.17–1.27)	(1.01–1.10)		(0.94–1.06)	(0.94–1.11)	(1.00–1.29)	(1.15–1.59)
Model 2	2.07 ***	2.42 ***	2.02 ***	1.56 ***	1.25 ***	1.06 **	1 (ref)	0.99	1.01	1.1	1.27 **
	(1.57–2.72)	(2.27–2.58)	(1.93–2.12)	(1.49–1.62)	(1.20–1.30)	(1.02–1.11)		(0.93–1.05)	(0.93–1.09)	(0.96–1.25)	(1.07–1.50)
Model 3	0.62 **	1.12 **	1.19 ***	1.11***	1.04 *	1 (ref)	1.03	1.08 **	1.15 **	1.31 ***	1.49 ***
	(0.46–0.84)	(1.05–1.20)	(1.14–1.24)	(1.07–1.15)	(1.01–1.08)		(0.98–1.07)	(1.02–1.15)	(1.06–1.25)	(1.15–1.49)	(1.26–1.76)
Model 4	0.61 **	1.10 **	1.18 ***	1.11 ***	1.05 *	1 (ref)	1.02	1.07 **	1.14 **	1.29 ***	1.46 ***
	(0.46–0.82)	(1.03–1.18)	(1.13–1.24)	(1.07–1.16)	(1.01–1.09)		(0.98–1.07)	(1.02–1.14)	(1.05–1.24)	(1.13–1.46)	(1.24–1.73)
Men											
Model 3	0.64 **	1.14 *	1.16 **	1.09 *	1.06	1 (ref)	1.04	1.25 *	1.77 **	1.02	1.19
	(0.47–0.89)	(1.03–1.26)	(1.07–1.26)	(1.00–1.18)	(0.97–1.15)		(0.91–1.19)	(1.04–1.51)	(1.30–2.40)	(0.57–1.84)	(0.56–2.49)
Model 4	0.65 **	1.14 **	1.17 **	1.09 *	1.06	1 (ref)	1.04	1.25 *	1.76 **	1	1.18
	(0.47–0.89)	(1.03–1.26)	(1.08–1.27)	(1.01–1.18)	(0.98–1.16)		(0.91–1.19)	(1.03–1.51)	(1.30–2.39)	(0.56–1.80)	(0.56–2.49)
Women											
Model 3	0.65	1.01	1.16 ***	1.10 ***	1.03	1 (ref)	1.02	1.07 *	1.12 **	1.33 ***	1.51 ***
	(0.27–1.58)	(0.88–1.14)	(1.09–1.23)	(1.05–1.15)	(0.99–1.07)		(0.98–1.07)	(1.01–1.14)	(1.03–1.22)	(1.16–1.51)	(1.28–1.79)
Model 4	0.65	1	1.16 ***	1.10 ***	1.03	1 (ref)	1.02	1.07 *	1.12 **	1.32 ***	1.50 ***
	(0.27–1.58)	(0.88–1.14)	(1.09–1.23)	(1.05–1.15)	(0.99–1.07)		(0.98–1.07)	(1.01–1.13)	(1.03–1.22)	(1.16–1.51)	(1.27–1.78)
High FPG											
Total											
Model 1	5.78 ***	4.90 ***	3.76 ***	2.58 ***	1.69 ***	1.24 ***	1 (ref)	0.98	1.01	1.12	1.77 **
	(4.05–8.26)	(4.40–5.45)	(3.43–4.11)	(2.36–2.82)	(1.54–1.85)	(1.13–1.37)		(0.85–1.13)	(0.83–1.22)	(0.84–1.51)	(1.28–2.46)
Model 2	3.55 ***	3.26 ***	2.68 ***	2.05 ***	1.50 ***	1.20 **	1 (ref)	0.99	1.03	1.13	1.73 **
	(2.47–5.11)	(2.92–3.64)	(2.44–2.95)	(1.87–2.24)	(1.37–1.65)	(1.08–1.32)		(0.86–1.14)	(0.85–1.25)	(0.84–1.52)	(1.25–2.40)
Model 3	0.83	1.26 **	1.34 ***	1.27 ***	1.14 **	1.07	1 (ref)	1.09	1.2	1.39 *	2.04 ***
	(0.56–1.24)	(1.11–1.42)	(1.21–1.48)	(1.16–1.40)	(1.04–1.26)	(0.97–1.18)		(0.94–1.25)	(0.99–1.46)	(1.03–1.87)	(1.47–2.84)

*p < 0.05, ** *p* < 0.01, *** *p* < 0.0001. Model 1: Unadjusted. Model 2: Adjusted for age and sex. Model 3: Model 2 plus adjustment for the use of anti-hypertensive therapy, history of cardiovascular disease, and habitual exercise (≥30 min exercise per session, >2 times/week vs. less frequent exercise), body mass index, systolic blood pressure, triglyceride, and smoking status. Model 4: Model 3 plus adjustment for the combined effect of sex, body mass index, exercise, and smoking status. High HbA_1c_—HbA_1c_ ≥ 42 mmol/mol (≥6.0%); high FPG—FPG ≥ 110 mg/dL (6.1 mmol/L).

## Supplementary Materials

The following are available online at https://www.mdpi.com/2077-0383/8/11/1825/s1, Table S1: Characteristics of male participants according to HDL-C category (*n* = 57,789), Table S2: Characteristics of female participants according to HDL-C category (*n* = 119,245).
