# Supplementary material for: Impaired Glucose Metabolism in People with Extremely Elevated High-Density Lipoprotein Cholesterol and Low Alcohol Consumption: Results of the Kanagawa Investigation of Total Checkup Data from the National Database-3 (KITCHEN-3)
Source: J Clin Med. 2019 Nov 1;8(11):1825. doi: 10.3390/jcm8111825 (PMC6912278; doi:10.3390/jcm8111825)
Supplement: Supplementary file 1 [file jcm-08-01825-s001.pdf]

Table S1. Characteristics of male participants according to HDL-C category (n = 57,789).

HDL-C category (mg/dL)	20–29	30–39	40–49	50–59	60–69	70–79	80–89	90–99	100–109	110–119	≥ 120
N (% of total)	213 (0.4)	5393 (9.3)	16632 (28.8)	17184 (29.7)	10586 (18.3)	4813 (8.3)	1872 (3.2)	755 (1.3)	223 (0.4)	78 (0.13)	40 (0.07)
s-Age (years)	55.1 ± 10.9	53.8 ± 10.2	53.6 ± 10.2	53.9 ± 10.3	54.4 ± 10.5	55.1 ± 10.6	56.0 ± 10.5	55.7 ± 10.5	56.7 ± 10.5	55.7 ± 10.6	56.9 ± 9.3
BMI (kg/m^2)	25.1 ± 2.9	24.8 ± 2.7	24.2 ± 2.7	23.3 ± 2.7	22.3 ± 2.7	21.6 ± 2.6	21.1 ± 2.5	20.6 ± 2.4	20.3 ± 2.5	20.1 ± 2.2	21.0 ± 2.6
SBP (mmHg)	123 ± 17.5	122 ± 15.7	122 ± 16.1	121 ± 16.2	120 ± 16.0	119 ± 16.2	119 ± 16.0	118 ± 15.5	121 ± 16.8	118 ± 16.9	121 ± 16.8
DBP (mmHg)	76.0 ± 11.5	76.6 ± 11.2	76.6 ± 11.2	75.8 ± 11.3	74.9 ± 11.1	74.2 ± 10.9	74.0 ± 11.0	73.5 ± 10.3	75.7 ± 11.0	74.8 ± 12.0	75.7 ± 10.8
HDL-C (mg/dL)	27 ± 1.9	36 ± 2.4	45 ± 2.8	54 ± 2.9	64 ± 2.8	74 ± 2.8	84 ± 2.8	94 ± 2.8	104 ± 2.7	114 ± 3.0	137 ± 16.1
LDL-C (mg/dL)	107 ± 33	129 ± 33	137 ± 32	134 ± 32	129 ± 31	126 ± 29	123 ± 28	119 ± 28	123 ± 29	120 ± 35	100 ± 38
TG, IQ (mg/dL)	235 (146-351)	169 (122-237)	124 (91-169)	95 (72-129)	78 (60-104)	69 (54-89)	63 (51-82)	59 (47-76)	61 (48-74)	59 (46-74)	65 (51-87)
HbA1c (%)	5.97 ± 0.62 [a]	5.98 ± 0.55 [a]	5.94 ± 0.50 [a]	5.88 ± 0.45 [a]	5.85 ± 0.39	5.82 ± 0.37	5.82 ± 0.36	5.82 ± 0.32	5.86 ± 0.31	5.83 ± 0.32	5.92 ± 0.48
FPG (mg/dL)	99.4 ± 19.3 [a]	98.4 ± 16.0 [a]	97.5 ± 14.4 [a]	96.1 ± 13.3 [a]	95.1 ± 11.8 [a]	94.4 ± 11.4	94.0 ± 10.7	93.7 ± 10.1	94.4 ± 11.1	92.5 ± 9.5	98.4 ± 14.6
Pharmacotherapy for hypertension, n (%)	30 (14.1)	802 (14.9)	2310 (13.9)	2042 (11.9)	1059 (10.0)	436 (9.1)	154 (8.2)	64 (8.5)	24 (10.8)	— [b]	— [b]
CVD, n (%)	— [b]	147 (2.7)	460 (2.8)	444 (2.6)	295 (2.8)	145 (3.0)	59 (3.2)	16 (2.1)	— [b]	— [b]	— [b]
Current smoking, n (%)	103 (48.4)	2388 (44.3)	5829 (35.1)	4495 (26.2)	2151 (20.3)	700 (14.5)	232 (12.4)	71 (9.4)	22 (9.9)	— [b]	— [b]
Habitual exercise, n (%) [c]	48 (22.5)	1171 (21.7)	4033 (24.3)	4847 (28.2)	3287 (31.1)	1663 (34.6)	719 (38.4)	280 (37.1)	88 (39.5)	33 (42.3)	18 (45.0)
Mild to moderate physical activity, n (%) [d]	89 (43)	1887 (35.6)	6557 (41.1)	7376 (43.6)	4823 (46.2)	2320 (48.8)	974 (53.1)	384 (51.5)	122 (55.0)	46 (60.5)	26 (65.0)

[a] Statistically significant difference in HbA1c and FPG was tested using Dunnett test, compared with the HDL-C group 80–89 mg/dL. [b] Not expressed because of the small number (< 10), which could affect participants' confidentiality. [c] Habitual exercise to a light sweat for over 30 min per session, twice weekly. [d] Physical activity (walking, and so on) more than 1 hour per day (available n = 56,864). The s-age, a prevalence of regular exercise and physical activity more than 1 hour per day were higher in the higher HDL-C groups than in the lower groups (ANOVA and Cochran–Armitage; all $p < 0.0001$). BMI, blood pressures, TG, LDL-C, HbA1c, and FPG were lower (ANOVA, all $p < 0.0001$). Pharmacotherapy for hypertension and current smoking, but not past history of CVD, were less prevalent in the higher HDL-C groups (Cochran–Armitage test; both $p < 0.0001$).

Table S2. Characteristics of female participants according to HDL-C category (n = 119,245).

HDL-C category (mg/dL)	20–29	30–39	40–49	50–59	60–69	70–79	80–89	90–99	100–109	110–119	≥ 120
N (% of total)	26 (0.02)	1233 (1.0)	8138 (6.8)	20586 (17.3)	29659 (24.9)	27233 (22.8)	17622 (14.8)	8977 (7.5)	3758 (3.2)	1305 (1.1)	708 (0.6)
s-Age (years)	56.4 ± 10.9	58.7 ± 10.9	57.8 ± 10.9	57.2 ± 11.0	56.5 ± 10.9	56.3 ± 10.7	56.3 ± 10.4	56.6 ± 10.2	56.8 ± 9.9	57.5 ± 9.6	58.3 ± 9.7
BMI (kg/m^2)	23.6 ± 2.5	24.0 ± 3.4	23.6 ± 3.3	22.7 ± 3.2	21.8 ± 3.0	21.0 ± 2.7	20.4 ± 2.5	20.0 ± 2.4	19.7 ± 2.3	19.4 ± 2.2	19.5 ± 2.5
SBP (mmHg)	124 ± 16.8	125 ± 18.1	123 ± 17.8	121 ± 18.1	119 ± 17.8	117 ± 17.7	117 ± 17.7	116 ± 17.6	116 ± 17.6	117 ± 17.6	117 ± 17.6
DBP (mmHg)	73.8 ± 11.9	74.6 ± 10.8	73.9 ± 11.3	72.8 ± 11.2	71.5 ± 11.2	70.9 ± 11.0	70.6 ± 11.0	70.5 ± 10.9	70.2 ± 10.9	70.5 ± 10.8	70.9 ± 10.9
HDL-C (mg/dL)	26 ± 2.2	37 ± 2.3	46 ± 2.7	55 ± 2.8	65 ± 2.9	74 ± 2.9	84 ± 2.8	94 ± 2.8	104 ± 2.8	114 ± 2.8	130 ± 10.7
LDL-C (mg/dL)	92 ± 37	127 ± 36	137 ± 33	137 ± 34	133 ± 33	130 ± 31	128 ± 30	128 ± 30	128 ± 30	129 ± 31	126 ± 32
TG, IQ (mg/dL)	265 (112-486)	168 (122-242)	127 (92-174)	96 (71-129)	78 (59-103)	68 (53-89)	63 (49-80)	59 (47-75)	56 (45-71)	55 (44-70)	55 (44-69)
HbA1c (%)	5.84 ± 0.44	6.00 ± 0.51 [a]	5.96 ± 0.45 [a]	5.90 ± 0.41 [a]	5.84 ± 0.36 [a]	5.82 ± 0.33 [a]	5.80 ± 0.31	5.81 ± 0.31	5.81 ± 0.30	5.83 ± 0.28	5.85 ± 0.28 [a]
FPG (mg/dL)	99.8 ± 26.3 [a]	96.5 ± 14.1 [a]	94.5 ± 12.7 [a]	92.6 ± 11.6 [a]	91.3 ± 10.6 [a]	90.5 ± 9.6 [a]	90.0 ± 9.4	90.3 ± 9.4	90.3 ± 9.2	90.5 ± 9.3	91.0 ± 9.8
Pharmacotherapy for hypertension, n (%)	— [b]	269 (21.8)	1508 (18.5)	3202 (15.6)	3446 (11.6)	2694 (9.9)	1557 (8.8)	727 (8.1)	268 (7.1)	91 (7.0)	71 (10.0)
CVD, n (%)	— [b]	46 (3.7)	211 (2.6)	418 (2.0)	517 (1.7)	496 (1.8)	319 (1.8)	154 (1.7)	62 (1.7)	16 (1.2)	14 (2.0)
Current smoking, n (%)	— [b]	200 (16.2)	942 (11.6)	1800 (8.7)	2043 (6.9)	1472 (5.4)	769 (4.4)	310 (3.5)	126 (3.4)	39 (3.0)	13 (1.8)
Habitual exercise, n (%) [c]	— [b]	338 (27.4)	2230 (27.4)	5708 (27.7)	8357 (28.2)	7946 (29.2)	5335 (30.3)	2947 (32.8)	1198 (31.9)	479 (36.7)	269 (38.0)
Mild to moderate physical activity, n (%) [d]	— [b]	531 (43.4)	3547 (43.9)	9405 (46.1)	14126 (48.1)	13553 (50.2)	9093 (52.1)	4907 (55.1)	2049 (55.0)	734 (56.8)	408 (58.1)

[a] Statistically significant difference in HbA1c and FPG using Dunnett test, compared with the HDL-C group 80–89 mg/dL. [b] Not expressed because of the small number (< 10), which could affect participants' confidentiality. [c] Habitual exercise to a light sweat for over 30 min per session, twice weekly. [d] Physical activity (walking, and so on) more than 1 hour per day (available n = 118,180). The prevalence of regular exercise and physical activity more than 1 hour per day were higher in the higher HDL-C groups than in the lower groups (ANOVA and Cochran–Armitage; both $p < 0.0001$). The s-age, BMI, blood pressures, TG, LDL-C, HbA1c, and FPG were lower (ANOVA, all $p < 0.0001$). Pharmacotherapy for hypertension, current smoking, and past history of CVD were less prevalent in the higher HDL-C groups (Cochran–Armitage; all $p < 0.0001$).
